# Directed in Vitro Evolution of Therapeutic Bacteriophages: The Appelmans Protocol

**DOI:** 10.3390/v11030241

**Published:** 2019-03-11

**Authors:** Ben H. Burrowes, Ian J. Molineux, Joe A. Fralick

**Affiliations:** 1 Department of Immunology and Molecular Microbiology, Texas Tech University Health Sciences Center, 3601 4th Street, Lubbock, TX 79430, USA; benburrowes@live.com; 2 Roche Molecular Systems, 983 University Avenue B200, Los Gatos, CA 95032, USA; 3 Center for Infectious Disease, Department of Molecular Biosciences, The University of Texas at Austin, 1 University Station A5000, Austin, TX 78712, USA

**Keywords:** Appelmans, bacteriophage evolution, bacteriophage recombination, phage therapy, *Pseudomonas aeruginosa*, antibiotic resistance

## Abstract

The ‘Appelmans protocol’ is used by Eastern European researchers to generate therapeutic phages with novel lytic host ranges. Phage cocktails are iteratively grown on a suite of mostly refractory bacterial isolates until the evolved cocktail can lyse the phage-resistant strains. To study this process, we developed a modified protocol using a cocktail of three *Pseudomonas* phages and a suite of eight phage-resistant (including a common laboratory strain) and two phage-sensitive *Pseudomona aeruginosa* strains. After 30 rounds of selection, phages were isolated from the evolved cocktail with greatly increased host range. Control experiments with individual phages showed little host-range expansion, and genomic analysis of one of the broad-host-range output phages showed its recombinatorial origin, suggesting that the protocol works predominantly via recombination between phages. The Appelmans protocol may be useful for evolving therapeutic phage cocktails as required from well-defined precursor phages.

## 1. Introduction

The emergence and increasing prevalence of bacterial strains that are resistant to available antibiotics poses a serious threat to world health [1], which, according to the World Health Organization (WHO), is heading toward a post-antibiotic era when many common infections will no longer have a cure [2]. This imminent threat demands an evaluation of novel approaches toward treating antibiotic-refractory infections. One such approach that is being re-examined is bacteriophage (phage) therapy, the use of bacterial viruses to specifically target and clear bacterial infections [3,4,5,6]. The National Institute of Allergy and Infectious Diseases included phage therapy as one of seven strategic approaches to combat antimicrobial resistance [7]. Bacteria and their phages have been evolving for over three billion years, and although bacteria can become resistant to phages, the latter, unlike antibiotics, can also evolve to infect otherwise resistant bacteria. For phages to be successful therapeutically, it may be necessary to continually isolate new phages that infect refractory bacterial strains. Therapeutic phages are usually isolated from the environment using sources such as sewage, marine and fresh water, or even patient samples [8]. While such sources can offer a great diversity of phages from which to select, it is not always possible or efficient to isolate phages with the most clinically relevant host range.

One approach successfully used in the Republic of Georgia is to prepare large cocktails of phages that target a broad range of pathogenic bacteria [9]. When a pathogen is encountered that is refractory to the cocktail, a new phage that can grow on the resistant pathogen is added, or what is referred to as the “Appelmans protocol” is employed to “invigorate” the cocktail (pers. comm. Dr Z. Alavidze, see Appendix A). This protocol is based on an empirical liquid method of phage titration developed in the 1920s by Appelmans [9,10]. Despite its history and common use by several laboratories in countries of the former Soviet Union and Europe, the protocol has only recently been reported in Western journals [11], and the mechanism(s) by which it works has not been determined.

For a mixture of phages, two major genetic mechanisms by which one or more phages adapt to a bacterium that was refractory to the original cocktail are spontaneous mutation and recombination. Phage genomes are architecturally mosaics, with each individual genome representing a unique assemblage of individual exchangeable modules [12,13,14,15]. Mechanisms for generating such mosaics include homologous recombination at shared boundary sequences of modular junctions, illegitimate recombination in a non-specific sequence-directed process, and site-specific recombination [13,16,17]. A cocktail of phages applied to host strains susceptible to several members of the mixture offers an opportunity for recombination events that can generate more genetic diversity in the phage pool.

In the contemporary laboratory, host-range mutants are usually isolated using a single phage, either through spontaneous or induced mutations by selecting for those that can grow on the resistant bacterial strain, which often map to the tail fiber genes (e.g. [16,18,19,20,21,22,23]). However, in the Appelmans protocol, a phage cocktail consisting of multiple phages is grown iteratively on multiple separate hosts, including resistant bacteria, until the cocktail evolves the ability to lyse the entire culture. The bacterial strains are maintained from their original stocks and are kept separate throughout. Any mutant phage with the ability to propagate on a strain that is refractory to other members of the mixture will be selected as phages are pooled at the end of each round of selection.

As a proof-of-principle experiment aimed at examining the mechanism of host-range expansion, we designed a 96-well plate format Appelmans protocol using a three-phage cocktail to which a suite of seven clinical isolates were initially refractory by plaque analysis. After 30 rounds of selection, an individual phage was isolated that could grow on all seven clinical isolates. Multiple recombination events between two phages in the cocktail were seen, at least some of which likely conferred an expanded host range. Our results describe a relatively simple, straightforward method by which a phage cocktail can expand its host range without the addition of new genetic information.

## 2. Materials and Methods

### 2.1. Bacterial and Bacteriophage Strains

All bacterial and bacteriophage strains used are listed in Table 1. The phages chosen for the protocol were ΦKZ, a large, virulent member of the *Myoviridae* family [24,25], Pa2, shown here to be an *N4virus* and used in our laboratory in previous in vivo studies of phage therapy [26], and phage RWG, also shown here to be an *N4virus,* which was isolated in our laboratory from a patient wound swab.

The bacterial strains included three common laboratory strains, PAO1, PA14 [27], and PAK [28]. PAO1 and PAK were kind gifts from Dr A. N. Hamood, Texas Tech University Health Sciences Center (TTUHSC); PA14 was generously donated by Dr K. Rumbaugh, TTUHSC. Seven clinical *Pseudomonas aeruginosa* strains were isolated from wound swabs within a six month period from patients at the Southwest Regional Wound Care Center (WCC), Lubbock, Texas. All isolates were from separate patients and showed high degrees of antibiotic resistance but with distinct antibiograms. These isolates are identified by the prefix WCC accompanied by a three-digit identifier (see Table 1). PAO1 was the fully permissive host used to propagate all *P. aeruginosa* phages.

Bacterial strains were grown in LB (5 g/L NaCl (Sigma Aldrich, St. Louis, MO, USA), 5 g/L yeast extract (BD, Franklin Lakes, NJ, USA), 10 g/L tryptone (BD, Franklin Lakes, NJ, USA)) supplemented with 1 mM CaCl_2_ (Sigma Aldrich, St. Louis, MO, USA) and 1 mM MgSO_4_ (Sigma Aldrich, St. Louis, MO, USA). Phages RWG and Pa2 were grown by infecting PAO1 at an A_600_ ~0.2, using an MOI ~0.1. After 3–5 h incubation at 37 °C, 250 rpm, the cultures were seen to lyse, and the phage was harvested by adding 1:100 CHCl_3_ (Sigma Aldrich, St. Louis, MO, USA), vortexing vigorously, and then centrifuging (≥18,000× *g*, 5 min, 4 °C) to remove cellular debris. The supernatants were stored over CHCl_3_ at 4 °C. ΦKZ was grown on semi-solid media as described elsewhere [25].

Where required, phage stocks were concentrated by the addition of 1/5 volume of 25% polyethylene glycol (PEG) M.W. 8000 (Sigma Aldrich, St. Louis, MO, USA), 2.5 M NaCl after incubation overnight at 4 °C. After centrifugation at 15,000× *g* for 15 min, the pellet was suspended in SM buffer (50 mM Tris-HCl [pH 7.5] (Sigma Aldrich, St. Louis, MO, USA), 0.1 M NaCl, 8 mM MgSO_4_, 0.01% *w*/*v* gelatin (Sigma Aldrich, St. Louis, MO, USA)). PEG precipitation was performed twice to give a final purified phage suspension [29]. Phage titers were assayed by the agar overlay method [30].

### 2.2. Analysis of Host Range

The Appelmans protocol was carried out as described in Results. Every 10 rounds, the pooled lysates were checked for novel phages by streaking a loopful (~1 µL) onto an LB agar plate and overlaying with ~10^7^ exponential-phase bacteria in top agar (LB plus 5 g/L agar). Five plaques were picked on each bacterial strain, choosing distinct plaque morphologies where possible, and were purified on the same strain. After at least three rounds of purification, the plaques were picked with a sterile Pasteur pipet and added to 2 mL early log-phase cells in supplemented LB. After lysis, or after 5 h incubation (whichever came first), the bacteria were killed by the addition of 20 µL CHCl_3_, and cell debris was removed by centrifugation at 15,000× *g* for 10 min. Most (38/50) phages isolated after the final round of Appelmans selection gave only faint, diffuse plaques and poor titers after purification and amplification on either the isolation strain or on PAO1; these phages were not studied further.

Host range was initially assessed by placing a 10 µL drop of phage suspension (≥10^6^ plaque-forming units, pfu) onto a pre-seeded lawn of host cells in top agar [30]. After overnight incubation at 37 °C, zones of lysis or the presence of plaques indicated that the bacterial strain was susceptible to the phage. The lytic host range of phage suspensions was further confirmed by titering to observe individual plaques.

### 2.3. DNA Sequencing and Annotation

The phages Pa2, RWG, and phi176 were purified by equilibrium density gradient centrifugation in CsCl [29]. Genomic DNA was isolated by extraction with phenol [31] and was subjected to 454 pyrosequencing by the University of Texas at Austin genomics core facility. Assembly used Newbler 2.6 and DNAStar software, followed by manual inspection of read frequency that revealed the presence of terminal repeats. These were taken as the physical genome ends but they were not formally established as such experimentally. Alignment of the Pa2, RWG, and phi176 genomes was performed using the NCBI Blast suite (https://blast.ncbi.nlm.nih.gov/Blast.cgi) and DNAStar. Genome sequences have been deposited in GenBank with Accession Numbers KM411958 (RWG), KM411959 (Pa2), and KM411960 (phi176).

The DNA sequences of phi176, Pa2, and RWG were annotated using the RAST (Rapid Annotation using Subsystem Technology) annotation service available at http://rast.nmpdr.org/ and manually. Differences were resolved by comparison to the previously annotated, closely related phage genomes PA26 (JX194238) and LIT1 (NC_013692) and, post facto, by internal comparisons. Open reading frames (ORFs) were analyzed for putative function using the Translated BLAST (blastx) and Protein BLAST (blastp) algorithms, searching the non-redundant protein sequences database.

### 2.4. Mitomycin C Induction and Southern Blot Analysis of Temperate Phages

Bacterial strains were grown to an A_600_ ~0.5 in LB before adding 4 µg/mL mitomycin C and incubating overnight. The cultures were then centrifuged and filtered through a 0.2 µM membrane. The resulting lysate was DNase I-treated prior to DNA extraction. Output phage DNA was digested with HincII and EcoRV (NEB), electrophoresed, and hybridized to a nitrocellulose membrane. Probes were prepared with temperate phage DNA using the ThermoFisher North2South Random Prime DNA Biotinylation Kit. Hybridization was detected using the ThermoFisher North2South Chemiluminescent Hybridization and Detection Kit.

## 3. Results

### 3.1. Appelmans ProtocolUusing a Phage Cocktail Expands Bacteriophage Host Ranges

**Figure 1:**
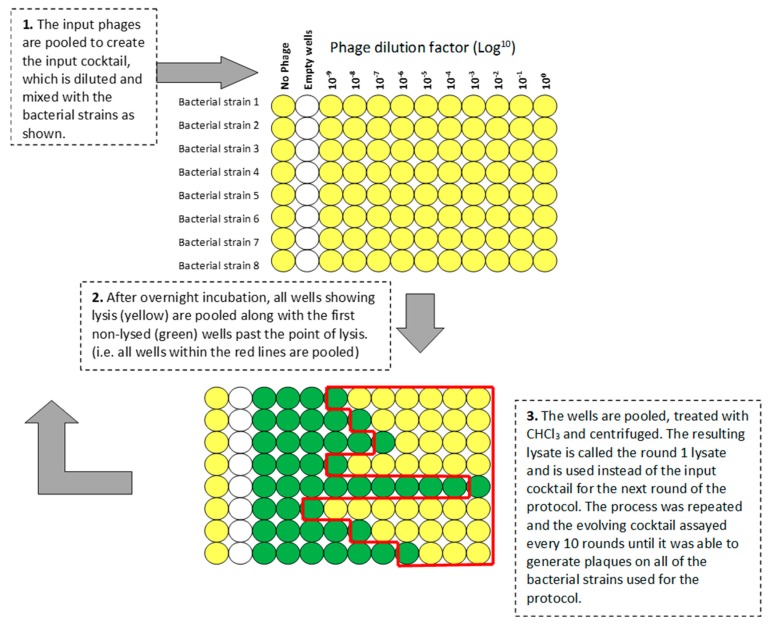
Schematic representation of the 96-well-plate-format Appelmans protocol.

In order to generate novel phages from our laboratory strains, we developed a protocol based on that used by the George Eliava Institute of Bacteriophage, Microbiology and Virology (IBMV), Tbilisi, Georgia (see Appendix A). The protocol is represented schematically in Figure 1. Phages Pa2, RWG, and ΦKZ were combined 1:1:1 by titer to yield an input cocktail of 1 × 10^10^ pfu/mL. Using a 96-well microtiter plate, 100 µL of serial 10-fold dilutions (10^0^ to 10^−9^) of the cocktail were added to 100 µL of double-strength LB containing 1 µL of an overnight bacterial culture of a single strain. Eight bacterial strains can be tested on each plate. One well was used for each dilution plus a control well with no phage. After overnight incubation at 37 °C on a shaking platform at 200 rpm, the plates were visually inspected. Wells showing complete lysis, plus the first turbid well, were pooled. If no lysis was seen, the well containing the undiluted phage cocktail was harvested. Pooled lysates were cleared by vortex mixing with 1:100 CHCl_3_, followed by centrifugation (15,000× *g* for 15 min). The lysate was termed the round 1 cocktail and was used to initiate the next round of the protocol using the same set of bacterial strains.

In the original Appelmans protocol the endpoint is reached when the output cocktail lyses >80% of the host strains at a minimum dilution of 10^−7^ (Appendix A). This cocktail is then usually used therapeutically. However, we were interested in analyzing the phages in the developing cocktail and we added additional steps in order to allow the analysis of individual phages present every 10 rounds of evolution. Table 2 shows that after 30 rounds, the evolved phage population generated plaques on all 10 *P. aeruginosa* strains, whereas the parent phages were only able to plaque on two laboratory strains.

The round-30 cocktail was plated onto each of the 10 bacterial strains used for the Appelmans protocol; five phages were isolated on each bacterial strain. For each plaque on a particular host, the same strain was used for further phage purification. Of the 15 plaques isolated on the laboratory *P. aeruginosa* strains PAO1, PA14, and PAK, eight phages were successfully amplified to high titer (≥10^8^ pfu/mL) on their isolation strain. Only 4 of the 35 plaques isolated on the WCC clinical strains contained phages that grew to high titers in liquid culture, either on their isolation strain or on PAO1, so only these were used for further study. Table 3 shows the host range of individual phages isolated from the round-30 cocktail. Phi229.2, phi229.1, and phi176 had a relatively broad host range that encompass, respectively, 80%, 90%, and 100% of the tested strains. Because of resource constraints, we obtained sequence data only for the parental strains and phi176; the genetic origins of phages phi229.1 and phi229.2 are therefore unknown.

Plating efficiencies (Table 4) show that PAO1 is highly susceptible to all phages, whereas PA14, PAK, and WCC201 are far more resistant. This is not surprising, as Pa2 and RWG (and ΦKZ) had been routinely propagated on PAO1 prior to this study, and the cell surfaces of PAO1, PAK, and PA14 are known to be different [32,33]. Cell surface differences can affect phage sensitivity. It is interesting that phi201 plated reasonably efficiently on both PAO1 and PAK but poorly on PA14, suggesting that the phages within the developing cocktail could be adapting to different hosts. In several cases, efficiency of plating (EOP) values >>1 were seen when the isolated phages were titered on non-host strains. These phages were better adapted to the hosts than to the host on which they were isolated, yielding the highest EOP. In other words, although they grew on a given strain, they grew better on an alternative host (usually PAO1). Although these phages were propagated primarily on the more permissive host, their maintenance during the Appelmans protocol was also selected for by the less permissive host(s) present. In this way, the protocol was able to select for and propagate rare phage mutants as they arose.

### 3.2. Appelmans Protocol Using Single Phages Generates Little Host-Range Expansion

When a single phage was used for the Appelmans protocol, little expansion in host range was seen (Supplementary Table S1A–C). Only phage RWG adapted to utilize a new host, and the total host-range expansion of all three phages separately was far less than that generated when the phages were combined into a cocktail. We saw no plaques from the single-phage Appelmans protocol experiments on most clinical *P. aeruginosa* isolates. Thus, although each individual phage was under continuous selection to grow on a mixture of different hosts, spontaneous mutations alone were insufficient to generate much change in host range.

### 3.3. Phi176 is a Recombinant Derivative of Phages Pa2 and RWG

At the outset of this study, we had no information on phage RWG and limited data on Pa2. As a preliminary check to determine whether recombination with endogenous genetic material (e.g., prophage or bacterial genes) was likely to contribute to phage evolution, we used Southern analyses of Pa2, RWG, and ΦKZ DNA with phage DNA isolated after mitomycin C induction of all bacterial strains. No hybridization was detected.

The genome of ΦKZ is known (GenBank: AF399011.1), but the genomes of phi176, Pa2, and RWG were sequenced for this study. Phages Pa2 and RWG are closely related, showing an overall nucleotide sequence identity of >99%. Both phages are also closely related to phages PA26 (JX194238, 97% identity), LIT1 (NC_013692, 97% identity), vB_PaeP_C2-10_Ab09 (GenBank: NC_024140.1, 98% identity), and PEV2 (NC_031063.1, 98% identity). Pa2 and RWG are therefore *N4virus Podoviridae* [34,35].

The genome of phi176 (GenBank: KM411960.1) is derived exclusively from phages Pa2 (GenBank: KM411959.1) and RWG (GenBank: KM411958.1), with no ΦKZ-derived sequences. Remarkably, only a single missense mutation—a residue not found at the corresponding position in either Pa2 or RWG—was definitively identified across the phi176 genome. The absence of point mutations was unexpected because of the extensive phage growth (~150 cycles) that took place during the Appelmans protocol—a conservative estimate is five rounds of phage replication for each round of the protocol (assuming a burst size of 50 pfu/cell, a single phage will be amplified to ~3 × 10^8^ pfu in five rounds).

**Figure 2:**
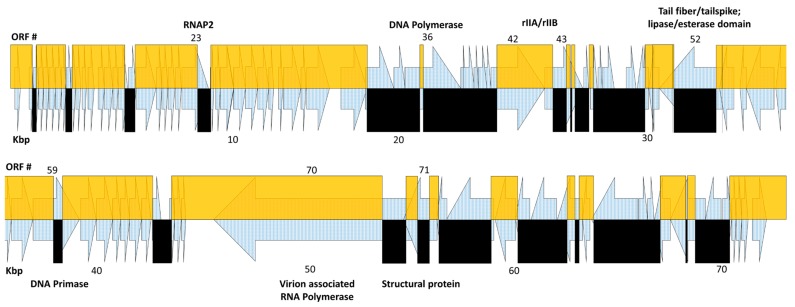
Schematic representation of the phi176 genome and its recombinatorial derivation. Open reading frame (ORF) numbers are shown at the top, and genome position (Kbp) at the bottom. Arrows represent individual ORFs. Yellow boxes denote genetic material of Pa2 origin, and black boxes represent RWG-derived sequences in the recombinant phi176 genome. The putative function of key recombinant genes (Table 5) are shown in bold above and below the map.

The Pa2, RWG, and phi176 genome sequences were aligned, allowing a visual determination that at least 48 crossovers occurred during the overall Appelmans procedure to yield phi176 (Figure 2 and Table 5). The terminal repeats of Pa2 and phi176 are the same, and the left (and right) genetic end of phi176 is thus derived from Pa2. Working in base-pair order (i.e., left to right), the first crossover from Pa2 DNA into RWG in generating phi176 is predicted by the last identical nucleotide to Pa2; this process was then repeated using the last position of identity on each respective genome over the complete sequence. Overall, about 2/3 of the phi176 genome is of Pa2 heritage. In Table 5, we named the gene by the crossover site predicted, but obviously, it is only known that recombination includes that site and not exactly where it was initiated or terminated. The phi176 sequence differed from both Pa2 and RWG at only five sites. One, at position 30,110 (phi176 ORF52) was a spontaneous missense mutation, but the remaining four (after phi176 positions 9355, 28671, 29,415, and 59342) occurred at homopolymeric runs and were judged more likely to be sequencing errors than mutational events. Two of the four affect intergenic regions, whereas the 151 amino acid phi176 ORF48 is only homologous to Pa2 and RWG for its initial 71 residues, and the 82 amino acid phi176 ORF50 differs in its last eight residues from both of its parents. The corresponding protein pairs from Pa2 and RWG were 100% identical and had no known or predicted function, supporting our premise of sequencing errors.

Forty-eight recombination events is a lower bound because, unless the recombination led to a change in sequence, it would go undetected; in addition, some recombination events might not have survived through all 30 rounds of the protocol. The majority (63/92) of ORFs underwent no crossovers between Pa2 and RWG in generating phi176. This was not unexpected because most genes are not directly involved in determining the host range. One recombination event was detected in 22 of the 29 recombinant ORFs, and one altered the ORF44/45 intergenic region (Table 5). Three ORFs underwent two crossovers, and a further three ORFs underwent three, whereas five and six recombination events occurred in ORF43 and ORF52, respectively. ORF52 also harbors the spontaneous missense mutation.

The frequencies of recombination within a given gene are not stochastic. Most ORFs undergoing multiple crossovers are somewhat larger than the average gene length of ~740 bp, but the putative phi176 virion RNA polymerase (ORF70), which is by far the largest gene at 10,197 bp, almost completely retained its Pa2 ancestry, undergoing only a single crossover across its length, despite ~275 polymorphisms with respect to its RWG counterpart. Retention of Pa2 DNA sequences in ORF70 is unlikely to be due to incompatibility of amino acid substitutions, as only 29 polymorphisms result in amino acid substitutions. Figure 2 shows the composition of the phi176 genome as recombinatorial crossovers between the Pa2 and RWG genomes, and Table 5 lists the Pa2 genes in which recombination occurred, generating phi176. Note that the phi176 rIIB-like protein ORF43 contained only two distinct amino acid substitutions, whereas six recombination events were detected within the gene; similarly, only four of six cross-overs in the phi176 ORF52 tailspike gene gave rise to amino acid substitutions. Recombination itself is of course totally distinct from any selective change conferred on an ORF by an amino acid substitution. However, it cannot be determined whether recombination events that did not lead to a change in an ORF were simply that, or whether they were fossils from prior events during the evolution of phi176.

## 4. Discussion

A cocktail comprised of three distinct phages was evolved over 30 rounds of the Appelmans protocol on a suite of seven clinical and three laboratory strains of *P. aeruginosa*. Of these 10 isolates, only two of the laboratory strains were sensitive to the original phages. When the protocol was carried out with each phage separately, very little expansion of host range was seen. However, after 30 rounds of the protocol with three input phages, progenies were isolated with expanded host ranges that included most (and in one case all) of the 10 bacterial strains. Genetic analysis of the phage with broadest host range showed it to be a recombinant derivative of the two most closely related members of the input cocktail: RWG and Pa2. These results support our premise that the Appelmans protocol is able to rapidly extend the host range of a cocktail of phages via recombination between the members of the cocktail.

The recombinant phage phi176 was essentially a result of multiple (≥48) recombination events between Pa2 and RWG, with only a single spontaneous point mutation. Given that ΦKZ is unrelated to Pa2 and that Pa2 and RWG share >99% DNA sequence identity, it is not surprising that no ΦKZ sequences were found in the phi176 recombinant. Although recombination between very different phages has clearly occurred in the environment (e.g., between the λ, P2, and T4 side-tail fibers [36], also see [12,13,14,15]), a short-term laboratory experiment provides little opportunity for recombination that does not involve significant DNA homology.

With so many recombination events occurring in genes of unknown function, it is impossible to elucidate exactly which of those event(s) were crucial in generating the host range of phi176. However, recombination clearly occurred in some genes likely to affect host range. The first step in infection is adsorption, which must involve structural gene products (e.g., tail fibers or tailspikes) that recognize a receptor on the surface of a bacterial cell. Two possible such gene products are encoded by phi176 ORF71 and ORF52. Although ORF71, which is a homologue of LIT1 gp72 [34], underwent three recombination events, it only differed from its Pa2 and RWG parents by a single amino acid residue. However, that was enough to confer host-range differences in both phages and eukaryotic viruses [19,21]. Phi176 ORF52, on the other hand, underwent six recombination events and additionally acquired a single point mutation. Furthermore, two of these recombinations resulted in a template switch for only three or seven contiguous nucleotides and hence were likely the end result of multiple recombination events in this region. Phi176 ORF52 differed from its parental sequences by 17 (RWG) and 75 (Pa2) amino acids. ORF52 is similar to LUZ7 gp56, or a hybrid of LIT1 gp52 and gp53, which have been tentatively identified as phage tail fibers [34]. However, as the protein sequences contain lipase and esterase motifs, they are more likely to be tailspikes. Phi176 ORF52 was therefore under strong selection for adaptation during growth on different host strains in the Appelmans protocol. Interestingly, phi176 ORF55, which putatively encodes a tail fiber, is not altered from its Pa2 ancestor. Perhaps, ORF55 is not required for phage adsorption on the strains tested here.

Resistance to phage is often achieved by cell surface receptor changes. Phages often respond by altering their receptor-binding protein(s), usually a tail fiber or tailspike. The most dramatic example is the accumulation of mutations found in Ox2 when selected to grow on a series of resistant strains. The tail fiber changed its receptor from OmpA, through other Omps, to LPS [22,23]. However, bacteria have several anti-phage defense mechanisms that operate after phage adsorption. These include CRISPR/Cas, restriction–modification systems, and other abortive infection mechanisms [37,38,39]. Some of these defenses are coded by prophage genes, including the exclusion of T4 *r*II mutants after infection of a *rex*+ λ lysogen [40]. T4 *r* mutants were originally isolated as plaque morphology variants on *Escherichia coli* B strains [41], which had lost the lysis inhibition characteristic of T4^+^ and thus exhibited a rapid-lysis phenotype [42]. However, this phenotype is not maintained in the non-lysogenic *E. coli* K-12 strains where plaques of *r*II mutants have wild-type morphology, and lysis inhibition is close to normal [40,43]. The rII proteins were originally only associated with T-even phages, but DNA sequencing has revealed similar proteins in the genomes of many different phage types, including the *N4virus* lineage and thus Pa2, RWG, and phi176, the phages studied here. It is important to note that only the roles of T4 *r*IIA and *r*IIB in overcoming the inhibitory effects of λ prophage *rexA* and *rexB* on T4 growth and the association of these genes with rapid lysis in *E. coli* B have been studied experimentally. Lysis inhibition, overcoming prophage- or plasmid-mediated exclusion or other functions of sequence homologues in other phages has not been directly demonstrated. Nevertheless, effects on lysis inhibition can alter plaquing host range because mechanisms that increase the rate of viral propagation compared to bacterial propagation, such as increasing burst size or reducing lysis time [37,44], can all increase fitness in vitro [45] and can lead to plaques from lysis-inhibition mutants when compared to their non-plaquing lysis-inhibited parents.

A single recombination event was seen in ORF42, a putative *r*IIA gene. The N-terminal halves of Pa2 and RWG ORF42 are very similar, and recombination between the two respective genes resulted in the phi176 ORF42 having just two amino acid changes from Pa2. The C-terminal half of phi176 ORF42 is derived entirely from Pa2, with 120 amino acids differing from its RWG counterpart. However, five recombination events took place in generating phi176 ORF43, a putative *r*IIB-like gene. The chimeric phi176 rIIB-like protein has 11 and 27 amino acid changes from ORF43 of RWG and Pa2, respectively. ORF43 was clearly under strong selection during adaptation in the Appelmans experiment to expand phage host range. By analogy with the T4 rII proteins, we suggest that phi176 ORFs 42 and 43 could counteract unknown inhibitory genes in the various clinical isolates that allowed for a phi176 host-range expansion, relative to either of its parents.

Directed evolution of phages is a common laboratory approach that has been used, among other things, to develop phages with enhanced therapeutic potential [46] and to study and modify phage host range [20,47,48,49]. We suggest that several factors appear to be crucial for the rapid generation of expanded host range phages with the Appelmans protocol. First, the use of a cocktail of virulent phages is critical to allow recombination to generate diversity within the protocol. When we isolated individual phages from the round-30 cocktail, those selected on our laboratory strains showed very little expansion of host range. The three phages with broadest host range we identified were isolated using bacterial strains refractory to the round-10 cocktail. However, when these broad-host-range phages were tested against a new set of urinary tract infection isolates of *P. aeruginosa*, isolated in a separate laboratory three years after the WCC isolates were obtained, no collateral expansion of host range was seen. Therefore, it appears that the use of both clinically relevant and up–to-date bacterial isolates that are resistant to the phages of the cocktail is important in generating therapeutically useful phages. It is noteworthy that the Eliava IBMV regularly adds new bacterial isolates to their Appelmans panel, and the phages generated are added to their complex and evolving therapeutic preparations (Pers. Comm., Z. Alavidze). We also believe it is important to include a bacterial host that can support lytic growth of all or most of the phages of the cocktail, thereby enabling recombination after co-infection. In contrast, collateral host-range expansion was maintained when a similar protocol was carried out by Mapes et al. [11], who saw an increase in host range on a set bacterial isolates that had not been used for phage evolution. It is not clear why the two studies arrived at different conclusions, and clearly, further work is needed both to optimize the Appelmans protocol and to better understand how to generate clinically useful phages efficiently.

Phage evolution is postulated to occur principally through recombination [12,13,15,17,50,51,52,53,54], and recombination has been demonstrated between the members of an experimental therapeutic cocktail of virulent *E. coli* phages in an anaerobic, continuous culture system [55]. Therefore, we consider it likely that phage evolution itself occurs predominantly through what is essentially the Appelmans protocol writ large. Indeed, the Appelmans protocol could already be occurring naturally, in situ, during active phage therapy [56,57,58]. Multiple rounds of infection and lysis in a dense and relatively localized bacterial population would provide many opportunities for horizontal exchange of genetic elements between therapeutic phages and even non-therapeutic phages present as prophages in the bacterial population or phages that are present in the local environment.

Our results are in keeping with this idea and suggest that recombination events between the members of a phage cocktail during the Appelmans protocol or during therapy generate far more genetic diversity than non-recombinant mutations alone. The total phenotypic diversity generated by all three single phage experiments was far less than that generated by evolving a cocktail of those same three phages. In the absence of recombination, we would have expected the total diversity generated from all three input phages separately to be similar to that generated by the three phages simultaneously. Moreover, only one point mutation was seen in the phi176 genome, whereas at least 48 recombination events occurred, corroborating the predominantly recombinatorial nature of phage evolution in the Appelmans protocol.

## Figures and Tables

**Table 1:** *Pseudomonas aeruginosa* and bacteriophage strains used for the Appelmans protocol. Plaque morphologies are described for growth on PAO1.

Bacterial/Phage Strain	Source	Reference	Notes
PAO1	Dr A. N. Hamood (TTUHSC)	[1]	Host strains used for the Appelmans protocol
PAK		[2]	
PA14	Dr K. Rumbaugh (TTUHSC)	[3]	
WCC176	Dr R. D. Wolcott MD Southwest Regional Wound Care Center. (Lubbock, TX)	This study	
WCC199			
WCC201			
WCC205			
WCC222			
WCC229			
WCC232			
Pa2	ATCC [14203-B1]	[1]	73,008 bp. Plaques 1–2 mm, clear
ΦKZ	Felix d’Herelle Reference Center for Viruses	[4]	280,334 bp. Plaques <1 mm, clear
RWG	This laboratory	This study	72,646 bp. Plaques 2–3 mm, clear

**Table 2:** Phage host range. A 10 μL spot of a lysate containing ≥ 10^6^ plaque-forming units (pfu) was placed onto an overlay lawn seeded with ~10^7^ colony-forming units (cfu) of *P. aeruginosa*. “+” indicates visible lysis after 16 h incubation at 37 °C, no entry indicates no visible lysis.

Test Strain	Phage					
	Pa2	ΦKZ	RWG	Round 10 Cocktail	Round 20 Cocktail	Round 30 Cocktail
PAO1	+	+	+	+	+	+
PA14				+	+	+
PAK	+	+		+	+	+
WCC176					+	+
WCC199				+	+	+
WCC201				+	+	+
WCC205				+	+	+
WCC222						+
WCC229						+
WCC232						+

**Table 3:** Host range of individual phages isolated from the round-30 cocktail.

Test Strain	Phage ^1^											
	phiPAO1.1	phiPAO1.2	phiPAO1.3	phiPA14.1	phiPA14.2	phiPAK.1	phiPAK.2	phiPAK.3	phi176	phi201	phi229.1	phi229.2
PAO1	+	+	+	+		+	+	+	+	+	+	+
PA14				+	+				+	+	+	+
PAK	+	+	+	+		+	+	+	+	+	+	+
WCC176									+		+	+
WCC199									+		+	
WCC201			+	+					+	+		
WCC205									+		+	+
WCC222									+		+	+
WCC229									+		+	+
WCC232									+		+	+

^1^ Phages are named with the prefix ‘phi’ followed by the isolation strain (see Table 1) and by the phage isolate number where appropriate (e.g., phi229.2 was the second phage isolated on strain WCC229).

**Table 4:** Efficiency of plating (EOP) and standard deviations (SD) of phages isolated on clinical isolates. ‘-’ indicates no plaques (<10 pfu/mL). EOP is determined as the phage titer on a test strain divided by the titer of the same phage preparation on the isolation host. EOP and SD values on the isolation strains are shown in bold.

Test Strain	phi176		phi201		phi229.1		phi229.2	
	EOP	SD	EOP	SD	EOP	SD	EOP	SD
PAO1	73.6	5.9	0.7	0.1	6.7	2.0	51.2	11.0
PA14	9.6 × 10^−5^	5.4 × 10^−4^	1.0 × 10^−9^	2.4 × 10^−8^	8.3 × 10^−5^	4.6 × 10^−5^	1.1 × 10^−3^	4.2 × 10^−4^
PAK	2.7 × 10^−5^	2.0 × 10^−6^	0.1	1.8 × 10^−2^	1.3 × 10^−6^	4.6 × 10^−7^	2.0 × 10^−4^	4.6 × 10^−5^
WCC176	**1.0**	**0.1**	-	-	1.5	0.5	7.9	2.7
WCC199	4.3	1.1	-	-	3.3 × 10^−2^	1.2 × 10^−2^	-	-
WCC201	1.8 × 10^−6^	1.2 × 10^−6^	**1.0**	**0.2**	-	-	-	-
WCC205	3.3	0.4	-	-	1.4	0.5	2.6	0.6
WCC222	18.2	1.6	-	-	1.1	0.3	2.6	0.8
WCC229	50.0	6.9	-	-	**1.0**	**0.4**	**1.0**	**0.3**
WCC232	39.3	6.7	-	-	0.1	4.7 × 10^−2^	7.9	1.9

**Table 5:** Recombinant phi176 ORFs.

Phi176 ORF	Start	Stop	ORF Size (bp)	Cross-Overs	Crossover Location(s) in phi176 Genome (bp) ^1^	Putative Gene Function
02	872	1063	192	1	1020	
03	1078	1308	231	1	1210	
08	2394	2597	204	1	2581	
10	2832	3065	234	1	2884	
17	5053	5493	441	1	5352	
18	5533	5880	348	1	5840	
23	8042	9283	1242	1	8764	RNA polymerase 2
23a	9380	9532	153	1	9384	
35	16,639	17,976	1167	1	16,721	DNA helicase
37	18,503	21,118	2616	2	19,203 19,359	DNA polymerase
42	22,534	25,050	2517	1	22,811	rIIA-like
43	25,062	26,840	1779	5	25,434 26,071 26,254 26,321 26,473	rIIB-like
IG44-45	N/A	N/A	N/A	1	27,137	N/A
45	27,196	27,513	318	1	27,326	
51	29,943	29,464	480	1	29,758	
52	33,215	29,943	3273	6 (+ 1 missense)	30,115 30,122 31,070 31,073 31,118 33,095	Tail fiber/tailspike; lipase/esterase domain
53	33,925	33,254	672	2	33,375 33,379	
59	37,720	39,888	2169	2	38,669 39,095	DNA primase P4 type
66	43,308	43,712	405	1	43,324	
67	44,118	44,354	237	1	44,210	
70	55,093	44,897	10,197	1	54,100	Virion RNA polymerase
71	56,659	55,094	1566	3	55,210 55,751 56,313	Structural protein
72	57,126	56,659	468	1	56,747	
73	59,329	57,107	2223	1	59,203	
75	61,019	60,354	666	1	60,455	
77	63,503	62,310	1194	3	62,768 63,143 63,333	
79	66,091	63,911	2181	1	64,015	
82	67,614	66,880	735	1	67,134	
83	69,263	67,611	1653	3	68,333 68,417 68,774	
85	70,055	70,486	432	1	70,398	

^1^ The starting 1–1020 bp and final 70,399–73,050 bp (including the terminal repeats) are derived from parental phage Pa2.

## Supplementary Materials

The following are available online at https://www.mdpi.com/1999-4915/11/3/241/s1, Table S1: Host range of single phage experiments.

## Appendix A

The Appelmans protocol, as supplied by Dr Z. Alavidze, IBMV. (In Georgia the protocol is often called the ‘Appelman’ technique.)

“The Appelman technique is a method for developing phage isolates into strains that are much more active against a wide range of bacterial strains of the same family. This long-standing technique is a main method used in the development of phage cocktails in the Republic of Georgia. The phage cocktail, generally active on at least 60% of the host collection, is diluted serially, with one dilution series for each of the 10–12 strains of interest, adding 0.1 mL of bacteria, diluted 10-fold from a slant tube, to each tube of 5 mL of broth. The tubes are incubated at 37 °C overnight. The following day, each dilution is checked for lysis compared to a control tube. The lysed tubes are combined across all the strains and filtered. The new filtrate is considered Appelman 1 and is used for the next dilution series, with the addition of one dilution past the previous point of lysis. Once a phage is able to lyse at a dilution of 10^−7^ across 10 or more strains, it is considered a “mother” phage that can be used in combination with other Appelman phages for a cocktail.

Qualitative analysis of the Appelman phages by spot testing shows an increase in virulence of the phage between the first and last Appelman. The efficiency of plating of each phage on the various strains is necessary to determine quantitatively how much change occurs as a result of Appelman passaging. The important question raised by this method is: how are the phages becoming more active? It is probable that a small change in the tail fibers of the phages allows for a better infection.”

## References

[1] Fauci A.S., Marston H.D. (2014). The Perpetual Challenge of Antimicrobial Resistance. JAMA.

[2] Smith R.D., Coast J. (2002). Antimicrobial resistance: A global response. Bull. World Health Organ..

[3] Burrowes B., Harper D.R., Anderson J., McConville M., Enright M.C. (2011). Bacteriophage therapy: Potential uses in the control of antibiotic-resistant pathogens. Expert Rev. Anti-Infect. Ther..

[4] Kutter E., De Vos D., Gvasalia G., Alavidze Z., Gogokhia L., Kuhl S., Abedon S.T. (2010). Phage therapy in clinical practice: Treatment of human infections. Curr. Pharm. Biotechnol..

[5] Abedon S.T. (2010). Hot Topic: The ‘Nuts and Bolts’ of Phage Therapy. Curr. Pharm. Biotechnol..

[6] Sulakvelidze A., Alavidze Z., Morris J.G. (2001). Bacteriophage therapy. Antimicrob. Agents Chemother..

[7] National Institutes of Health (2014). NIAID’s Antibacterial Resistance Program: Current Status and Future Directions.

[8] Gill J.J., Hyman P. (2010). Phage choice, isolation, and preparation for phage therapy. Curr. Pharm. Biotechnol..

[9] Chanishvili N., Sharp R. (2009). Eliava Institute of Bacteriophage, Microbiology and Virology, Tbilisi, Georgia. A Literature Review of the Practical Application of Bacteriophage Research.

[10] Appelmans R. (1921). Le dosage du Bacteriophage. Compt. Rend. Soc. Biol..

[11] Mapes A.C., Trautner B.W., Liao K.S., Ramig R.F. (2016). Development of expanded host range phage active on biofilms of multi-drug resistant Pseudomonas aeruginosa. Bacteriophage.

[12] Hendrix R.W. (2003). Bacteriophage genomics. Curr. Opin. Microbiol..

[13] Hendrix R.W. (2002). Bacteriophages: Evolution of the majority. Theor. Popul. Biol..

[14] Krylov V., Pleteneva E., Bourkaltseva M., Shaburova O., Volckaert G., Sykilinda N., Kurochkina L., Mesyanzhinov V. (2003). Myoviridae bacteriophages of Pseudomonas aeruginosa: A long and complex evolutionary pathway. Res. Microbiol..

[15] Silander O.K., Weinreich D.M., Wright K.M., O’Keefe K.J., Rang C.U., Turner P.E., Chao L. (2005). Widespread genetic exchange among terrestrial bacteriophages. Proc. Natl. Acad. Sci. USA.

[16] Tetart F., Desplats C., Krisch H. (1998). Genome plasticity in the distal tail fiber locus of the T-even bacteriophage: Recombination between conserved motifs swaps adhesin specificity. J. Mol. Biol..

[17] Morris P., Marinelli L.J., Jacobs-Sera D., Hendrix R.W., Hatfull G.F. (2008). Genomic characterization of mycobacteriophage Giles: Evidence for phage acquisition of host DNA by illegitimate recombination. J. Bacteriol..

[18] Tétart F., Repoila F., Monod C., Krisch H. (1996). Bacteriophage T4 Host Range Is Expanded by Duplications of a Small Domain of the Tail Fiber Adhesin.

[19] Shioda T., Levy J.A., Cheng-Mayer C. (1992). Small amino acid changes in the V3 hypervariable region of gp120 can affect the T-cell-line and macrophage tropism of human immunodeficiency virus type 1. Proc. Natl. Acad. Sci. USA.

[20] Morona R., Henning U. (1984). Host range mutants of bacteriophage Ox2 can use two different outer membrane proteins of Escherichia coli K-12 as receptors. J. Bacteriol..

[21] Werts C., Michel V., Hofnung M., Charbit A. (1994). Adsorption of bacteriophage lambda on the LamB protein of Escherichia coli K-12: Point mutations in gene J of lambda responsible for extended host range. J. Bacteriol..

[22] Drexler K., Riede I., Montag D., Eschbach M.-L., Henning U. (1989). Receptor specificity of the Escherichia coli T-even type phage Ox2: Mutational alterations in host range mutants. J. Mol. Biol..

[23] Drexler K., Dannull J., Hindennach I., Mutschler B., Henning U. (1991). Single mutations in a gene for a tail fiber component of an Escherichia coli phage can cause an extension from a protein to a carbohydrate as a receptor. J. Mol. Biol..

[24] Kwan T., Liu J., DuBow M., Gros P., Pelletier J. (2006). Comparative genomic analysis of 18 Pseudomonas aeruginosa bacteriophages. J. Bacteriol..

[25] Mesyanzhinov V., Robben J., Grymonprez B., Kostyuchenko V., Bourkaltseva M., Sykilinda N., Krylov V., Volckaert G. (2002). The genome of bacteriophage phiKZ of Pseudomonas aeruginosa. J. Mol. Biol..

[26] McVay C.S., Velásquez M., Fralick J.A. (2007). Phage therapy of Pseudomonas aeruginosa infection in a mouse burn wound model. Antimicrob. Agents Chemother..

[27] Rahme L.G., Stevens E.J., Wolfort S.F., Shao J., Tompkins R.G., Ausubel F.M. (1995). Common virulence factors for bacterial pathogenicity in plants and animals. Science.

[28] Takeya K., Amako K. (1966). A rod-shaped Pseudomonas phage. Virology.

[29] Carlson K. (2005). Appendix: Working with Bacteriophages: Common Techniques and Methodological Approaches. Bacteriophages: Biology and applications.

[30] Adams M. (1959). Enumeration of bacteriophage particles. Bacteriophages.

[31] Sambrook J., Russel D.W. (2006). Extraction of Bacteriophage λ DNA from Large-scale Cultures Using Proteinase K and SDS. Cold Spring Harb. Protoc..

[32] ParaBioSys. https://pga.mgh.harvard.edu/Parabiosys/projects/host-pathogen_interactions/sequencing.php.

[33] Sastry P., Finlay B.B., Pasloske B., Paranchych W., Pearlstone J., Smillie L. (1985). Comparative studies of the amino acid and nucleotide sequences of pilin derived from Pseudomonas aeruginosa PAK and PAO. J. Bacteriol..

[34] Ceyssens P.-J., Brabban A., Rogge L., Lewis M.S., Pickard D., Goulding D., Dougan G., Noben J.-P., Kropinski A., Kutter E. (2010). Molecular and physiological analysis of three Pseudomonas aeruginosa phages belonging to the “N4-like viruses”. Virology.

[35] Ceyssens P.J., Noben J.P., Ackermann H.W., Verhaegen J., De Vos D., Pirnay J.P., Merabishvili M., Vaneechoutte M., Chibeu A., Volckaert G. (2009). Survey of Pseudomonas aeruginosa and its phages: De novo peptide sequencing as a novel tool to assess the diversity of worldwide collected viruses. Environ. Microbiol..

[36] Haggård-Ljungquist E., Halling C., Calendar R. (1992). DNA sequences of the tail fiber genes of bacteriophage P2: Evidence for horizontal transfer of tail fiber genes among unrelated bacteriophages. J. Bacteriol..

[37] Labrie S.J., Samson J.E., Moineau S. (2010). Bacteriophage resistance mechanisms. Nat. Rev. Microbiol..

[38] Labrie S.J., Moineau S. (2007). Abortive infection mechanisms and prophage sequences significantly influence the genetic makeup of emerging lytic lactococcal phages. J. Bacteriol..

[39] Samson J.E., Magadán A.H., Sabri M., Moineau S. (2013). Revenge of the phages: Defeating bacterial defences. Nat. Rev. Microbiol..

[40] Benzer S. (1955). Fine structure of a genetic region in bacteriophage. Proc. Natl. Acad. Sci. USA.

[41] Hershey A.D. (1946). Mutation of bacteriophage with respect to type of plaque. Genetics.

[42] Doermann A.H. (1948). Lysis and lysis inhibition with Escherichia coli bacteriophage. J. Bacteriol..

[43] Burch L.H., Zhang L., Chao F.G., Xu H., Drake J.W. (2011). The bacteriophage T4 rapid-lysis genes and their mutational proclivities. J. Bacteriol..

[44] Abedon S.T., Yin J. (2009). Bacteriophage plaques: Theory and analysis. Bacteriophages.

[45] Nguyen A.H., Molineux I.J., Springman R., Bull J.J. (2012). Multiple genetic pathways to similar fitness limits during viral adaptation to a new host. Evolution.

[46] Merril C.R., Biswas B., Carlton R., Jensen N.C., Creed G.J., Zullo S., Adhya S. (1996). Long-circulating bacteriophage as antibacterial agents. Proc. Natl. Acad. Sci. USA.

[47] Abe M., Izumoji Y., Tanji Y. (2007). Phenotypic transformation including host-range transition through superinfection of T-even phages. FEMS Microbiol. Lett..

[48] Yoichi M., Abe M., Miyanaga K., Unno H., Tanji Y. (2005). Alteration of tail fiber protein gp38 enables T2 phage to infect Escherichia coli O157: H7. J. Biotechnol..

[49] Meyer J.R., Dobias D.T., Weitz J.S., Barrick J.E., Quick R.T., Lenski R.E. (2012). Repeatability and contingency in the evolution of a key innovation in phage lambda. Science.

[50] Brüssow H., Hendrix R.W. (2002). Phage genomics: Small is beautiful. Cell.

[51] Juhala R.J., Ford M.E., Duda R.L., Youlton A., Hatfull G.F., Hendrix R.W. (2000). Genomic Sequences of Bacteriophages HK97 and HK022: Pervasive Genetic Mosaicism in the Lambdoid Bacteriophages. J. Mol. Biol..

[52] Mmolawa P.T., Schmieger H., Heuzenroeder M.W. (2003). Bacteriophage ST64B, a genetic mosaic of genes from diverse sources isolated from Salmonella enterica serovar typhimurium DT 64. J. Bacteriol..

[53] Pajunen M.I., Elizondo M.R., Skurnik M., Kieleczawa J., Molineux I.J. (2002). Complete nucleotide sequence and likely recombinatorial origin of bacteriophage T3. J. Mol. Biol..

[54] Pedulla M.L., Ford M.E., Houtz J.M., Karthikeyan T., Wadsworth C., Lewis J.A., Jacobs-Sera D., Falbo J., Gross J., Pannunzio N.R. (2003). Origins of Highly Mosaic Mycobacteriophage Genomes. Cell.

[55] Kunisaki H., Tanji Y. (2010). Intercrossing of phage genomes in a phage cocktail and stable coexistence with Escherichia coli O157: H7 in anaerobic continuous culture. Appl. Microbiol. Biotechnol..

[56] Abedon S.T., Thomas-Abedon C. (2010). Phage therapy pharmacology. Curr. Pharm. Biotechnol..

[57] Payne R.J., Jansen V.A. (2000). Phage therapy: The peculiar kinetics of self-replicating pharmaceuticals. Clin. Pharmacol. Ther..

[58] Payne R.J., Jansen V.A. (2003). Pharmacokinetic principles of bacteriophage therapy. Clin. Pharmacokinet..

