# Supplementary material for: Directed in Vitro Evolution of Therapeutic Bacteriophages: The Appelmans Protocol
Source: Viruses. 2019 Mar 11;11(3):241. doi: 10.3390/v11030241 (PMC6466182; doi:10.3390/v11030241)
Supplement: Supplementary file 1 [file viruses-11-00241-s001.pdf]

Supplementary Table, A-C. Host range of single phage experiments. [a] Ap10, 20, 30 indicates the number of rounds of the MAP conducted. phiPa2.30, phiKZ.30 and phiΦRWG.30 are the broadest host range phages isolated after 30 rounds of development of each parental phage. +: lysis on the test strain, -: no lysis.

A

	Phage[a]				
Test strain	Pa2	Ap10.Pa2	Ap20.Pa2	Ap30.Pa2	phiPa2.30
PAO1	+	+	+	+	+
PA14	-	-	-	-	-
PAK	+	+	+	+	+
WCC176	-	-	-	-	-
WCC199	-	-	-	-	-
WCC201	-	-	-	-	-
WCC205	-	-	-	-	-
WCC222	-	-	-	-	-
WCC229	-	-	-	-	-
WCC232	-	-	-	-	-

B

	Phage[a]				
Test strain	ΦKZ	Ap10.ΦKZ	Ap20.ΦKZ	Ap30.ΦKZ	phiKZ.30
PAO1	+	+	+	+	+
PA14	-	-	-	-	-
PAK	+	+	+	+	+
WCC176	-	-	-	-	-
WCC199	-	-	-	-	-
WCC201	-	-	-	-	-
WCC205	-	-	-	-	-
WCC222	-	-	-	-	-
WCC229	-	-	-	-	-
WCC232	-	-	-	-	-

C

	Phage[a]				
Test strain	RWG	Ap10.RWG	Ap20.RWG	Ap30.RWG	phiRWG.30
PAO1	+	+	+	+	+
PA14	-	-	-	-	-
PAK	-	+	+	+	+
WCC176	-	-	-	-	-
WCC199	-	-	-	-	-
WCC201	-	-	-	+	+
WCC205	-	-	-	-	-
WCC222	-	-	-	-	-
WCC229	-	-	-	-	-
WCC232	-	-	-	-	-
